# Assessing regional disparities and trends in health resource allocation from sub-provincial cities in China

**DOI:** 10.1080/07853890.2025.2522962

**Published:** 2025-06-26

**Authors:** Minghua Zhou

**Affiliations:** Department of Administration Office, Luzhou People’s Hospital, Luzhou, Sichuan, China

**Keywords:** China, dynamic evolution, health resource allocation, regional differences, sub-provincial cities

## Abstract

**Background:**

To analyze the regional disparities and trends in health resource allocation in sub-provincial cities, to inform strategies for achieving equitable health resource allocation across sub-provincial cities.

**Methods:**

Dagum Gini coefficient and kernel density estimation based on the health resource density index were used to analyze the regional disparities and trends in health resource allocation in sub-provincial cities from 2018 to 2022.

**Results:**

The overall Gini coefficient of health expenditure is the highest, above 0.373. The differences in health expenditure are larger in the eastern region, and smaller in the central and western regions in terms of medical and health institutions, number of beds, health technicians, licensed (assistant) physicians and registered nurses. The inter-regional contribution rate is the main source of differences, with the lowest value of the inter-regional contribution rate being 45.08% of health expenditure in 2020 and the highest value being 67.60% of licensed (assistant) physicians in 2020. The longer and longer right trailing tail of the health expenditure kernel density curve indicates a significant difference in health expenditure. The kernel density curves of health expenditure and medical and health institutions have a multi-peaked distribution, indicating a low degree of equalization, while the rest of the distribution of health resources is relatively more balanced.

**Conclusion:**

Health resources in sub-provincial cities have continued to grow. Sub-provincial cities have a more balanced distribution of health resources, but there are significant differences in health expenditure. Inter-regional differences are the main cause of differences in health resource allocation in sub-provincial cities.

## Introduction

According to the administrative level, Chinese cities can be divided into municipalities, sub-provincial cities, prefectural cities and county-level cities. The implementation of sub-provincial cities, which began on February 25, 1994, is not only conducive to accelerating the economic and social development of the cities, but also to giving better play to the radiant role of the central cities. Sub-provincial cities have stronger administrative autonomy, partial legislative power and higher financial autonomy, and greater autonomy in the input and distribution of financial funds, with clear advantages in government investment, financial subsidies and resource allocation. The health resources of sub-provincial cities are better than those of ordinary cities in terms of both quantity and quality, but there are still problems such as the uneven distribution of health resources between cities and the insufficient supply of health resources in some cities. The construction of sub-provincial cities has a pivotal position, not only to build a good model of socio-economic development, but also to promote the development of other regions through its own radiation effect [1]. In April 2022, the Notice of the General Office of the State Council of China on the Issuance of the 14th Five-Year Plan for National Health (Guo Ban Fa [2022] No. 11) called for making it a priority to improve the quality of the supply of health services, accelerating the expansion of high-quality medical and health resources and the balanced layout of the region, and continually improving the equity and accessibility of basic medical and health services. Therefore, the study of regional disparities in the allocation of health resources in sub-provincial cities is not only conducive to promoting the regional balanced development of health resources in sub-provincial cities [2], but also helps to promote sub-provincial cities to give full play to the driving role of high-quality medical resources. Sub-provincial cities play a key role in balancing the allocation of medical resources and upgrading the quality and level of health services, thereby promoting the better utilization of high-quality health resources for the benefit of the people.

At present, studies on urban health resource allocation are available globally, mainly including estimates of geographic access to health facilities by urbanization degree in sub-Saharan Africa [3], urban–rural differences in health service utilization in upper-middle-income and high-income countries [4], access to specialty healthcare for urban versus rural populations in the United States [5], measures of the availability of human resources for health in 204 countries and territories [6], and urban–rural differences in health outcomes, health behaviors, and social determinants of health [7], etc. Specifically, studies on urban health resources and health services in China are divided into the following three main areas. First, studies related to city size and health care policy. It mainly included the effect of urban scale on health services inequity in China [8], the health impact of the urban expansion of small cities in China [9], and the evaluation of the health policy of the basic health insurance scheme of urban employees in China [10]. Second, studies related to the comparison of urban and rural health services. It mainly included the imbalance in the distribution of the primary health workforce between urban and rural areas in China [11], the measurement and influencing factors of the demand for care services of disabled elderly in urban and rural China [12], and the rural–urban differences in patient experience in China [13]. Third, studies related to the utilization of medical services and medical costs for urban patients. It mainly included the annual prevalence, medical service utilization and direct costs of lung cancer in urban China [14], the medical service utilization and direct medical costs of stroke in urban China [15], and the medical service utilization and direct medical costs of depression in urban China [16]. In addition, there are some empirical studies on the spatial allocation of health resources, mainly including the interprovincial spatial distribution pattern of Traditional Chinese Medicine service utilization in China [17], the evolution of the geographical pattern of health resources in China [18], and the spatial variations in health service utilization among migrant population in China [19]. In general, current studies on urban health resources in China mainly focus on the comparison of urban and rural health services and the utilization of medical services by urban patients, while studies on the regional differences and dynamic evolution of urban health resources, especially those in sub-provincial cities, need to be further deepened. What is the situation of health resource allocation in sub-provincial cities, and what is the process of dynamic evolution of health resources, these are important issues that deserve in-depth study. Through the study of regional disparities and trends in health resources in sub-provincial cities, proposed targeted measures to improve the allocation of health resources in sub-provincial cities, promoted the regional balanced development of health resources in sub-provincial cities, and facilitated sub-provincial cities to give full play to the driving role of high-quality medical resources, which will be conducive to promoting the overall health equity and regional development in China, and continuously improving the equity and accessibility of medical services.

In order to accurately recognize the regional disparities in health resources in sub-provincial cities and the direction of the next step of development, this study uses Dagum Gini coefficient and kernel density estimation based on the health resource density index to analyze the regional differences and dynamic evolution of health resource allocation in sub-provincial cities in China, and to inform strategies for achieving equitable health resource allocation across sub-provincial cities.

## Methods

### Data sources

Data on health resources in sub-provincial cities in China were obtained from the China Health Statistics Yearbook from 2019 to 2023, and some of the data were supplemented by the City Statistical Yearbook published by the statistical bureaus of each city. Individual data vary according to the year of release and are based on the latest year of release. Individual missing data are supplemented by interpolation according to the characteristics of the indicators and data trends. The scope of the study is the data on health resources in 15 sub-provincial cities in China from 2018 to 2022.

### Region division

**Figure 1.**
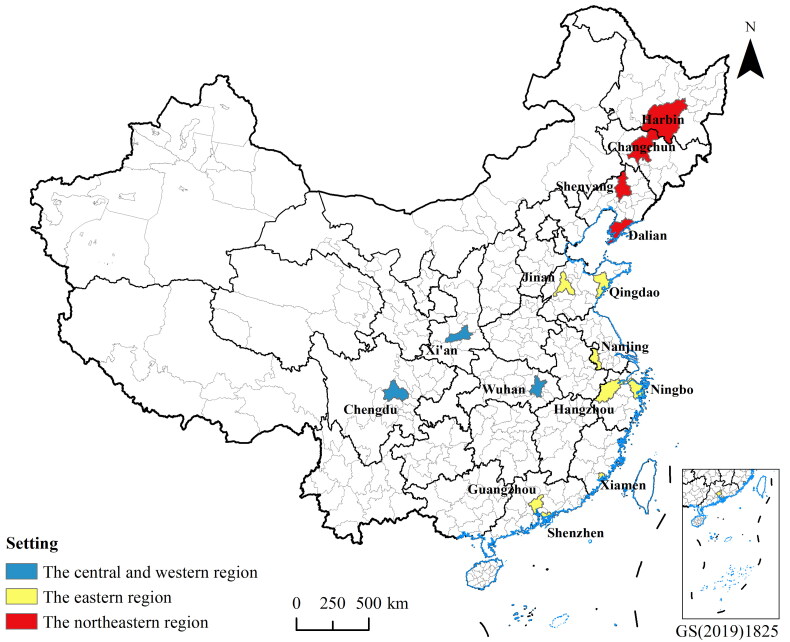
The distribution of 15 sub-provincial cities in China. The base map is derived from the national natural resources and geospatial basic information database of PRC (https://www.sgic.net.cn/portal/index.html#/home), review number GS(2019)1825, and these shapes are licensed without need for permission.

Sub-provincial cities are the second level of cities in China, and are prefecture cities where the administrative level of key leading cadres of the Party and government organs is the deputy provincial minister level, which was officially implemented on February 25, 1994. There are 15 sub-provincial cities in China, including Guangzhou, Wuhan, Harbin, Shenyang, Chengdu, Nanjing, Xi’an, Changchun, Jinan, Hangzhou, Dalian, Qingdao, Shenzhen, Xiamen and Ningbo. Among them, Shenzhen, Dalian, Qingdao, Ningbo and Xiamen are single-provincial cities, while the others are provincial capitals. According to the regional coordinated development strategy of accelerated development in the eastern region, development of the western region, revitalization of the northeastern region and the rise of central region, according to the province in which each sub-provincial city is located, the 15 sub-provincial cities are divided into three regions, the northeastern region, the eastern region, and the central and western region, among which the central region consists of only Wuhan, and the western region consists of only Chengdu and Xi’an, so that the central region and the western region are merged to form the central and western region. The northeastern region includes 4 cities, Changchun, Shenyang, Dalian, and Harbin; the eastern region includes 8 cities, Guangzhou, Nanjing, Jinan, Hangzhou, Qingdao, Shenzhen, Xiamen, and Ningbo; and the central and western region includes 3 cities, Wuhan, Chengdu, and Xi’an (Figure 1).

### Indicators

Health resources include health physical resources, health human resources and health financial resources [20], and 6 indicators, namely, medical and health institutions, number of beds, health technicians, licensed (assistant) physicians, registered nurses and health expenditure, are selected as evaluation indicators, among which medical and health institutions and number of beds represent health physical resources, health technicians, licensed (assistant) physicians and registered nurses represent health human resources, and health expenditure represent health financial resources.

### Research methods

Considering the impact of population and geographic factors on health resource allocation, the Dagum Gini coefficient based on health resource density index was used to analyze the regional differences in health resource allocation in sub-provincial cities in China, and the kernel density estimation based on health resource density index was used to analyze the dynamic evolution process of health resource allocation in sub-provincial cities in China.

### Health resource density index (HRDI)

Equitable and accessible allocation of health resources is the most basic and important principle. Existing studies have confirmed the feasibility of studying the allocation of health resources in terms of both population and geographic area, and the combination of population and geographic factors is more conducive to the allocation of health resources because of the existence of a vast geographic area with a sparse population, or a sparse geographic area with a vast population. The health resource density index is the geometric mean of the product of health resources per 1,000 population and health resources per square kilometer. The health resource density index is an important indicator that combines population and geographic factors in the allocation of health resources [21], taking into account not only the distribution of health service recipients, but also the accessibility of the population to health care. The higher the health resource density index, the better the allocation of health resources. The formula is:

$$\text{HRDI}=\sqrt{\frac{\text{Health resources}}{\text{Per}\;1,000\;\text{population}}\times \frac{\text{Health resources}}{\text{Per square kilometer}}}$$

### Dagum Gini coefficient

The Dagum Gini coefficient explores the issue of the sources of regional differences on the basis of the Gini coefficient, which decomposes the sources of regional differences into three components: intra-regional difference contribution, inter-regional difference contribution, and hypervariable density contribution [22]. The Dagum Gini coefficient effectively solves the problem that the traditional Gini coefficient cannot decompose regional differences and the Theil index cannot take into account the distribution of subsamples and the cross-over between samples, and it can better explain the problem of the source of regional differences [23]. A higher Dagum Gini coefficient indicates poorer equity. The Dagum Gini coefficient and its decomposition are calculated using the following formula:

$$G=\frac{\sum_{j=1}^{k}\sum_{h=1}^{k}\sum_{i=1}^{\text{nj}}\sum_{r=1}^{nh}\left|y_{\text{ji}}-y_{\text{hr}}\right|}{2n^{2}\bar{y}}$$

where *G* is the overall Gini coefficient, *k* is the number of regions, *n* is the number of cities, n_j_(n_h_) is the number of cities in region *j*(*h*), $y_{\text{ji}}$ ($y_{\text{hr}}$) is health resource density index for each city in the region, and $\bar{y}$ is the mean value of health resource density index for each city.

$$G=G_{w}+G_{\text{nb}}+G_{t}$$

where $G_{w}$ is the degree to which differences in distribution within region *j*(*h*) contribute to the overall Gini coefficient, $G_{\text{nb}}$ is the degree to which differences in distribution between regions *j* and *h* contribute to the overall Gini coefficient, and $G_{t}$ is the degree to which hypervariance density contributes.

$$G_{w}=\sum_{j=1}^{k}G_{\text{jj}}P_{j}S_{j}$$

$$G_{\text{nb}}=\sum_{j=2}^{k}\sum_{h=1}^{j-1}G_{\text{jh}}(P_{j}S_{h}+P_{h}S_{j})D_{\text{jh}}$$

$$G_{t}=\sum_{j=2}^{k}\sum_{h=1}^{j-1}G_{\text{jh}}(P_{j}S_{h}+P_{h}S_{j})(1-D_{\text{jh}})$$

$$G_{\text{jj}}=\frac{\frac{1}{2\bar{Y_{j}}}\sum_{i=1}^{n_{j}}\sum_{r=1}^{n_{j}}\left|y_{\text{ji}}-y_{\text{jr}}\right|}{n_{j}^{2}}$$

$$G_{\text{jh}}=\frac{\sum_{i=1}^{n_{j}}\sum_{r=1}^{n_{h}}\left|y_{\text{ji}}-y_{\text{hr}}\right|}{n_{j}n_{h}(\bar{Y_{j}}+\bar{y_{h}})}$$

where *P_j_ = n_j_ /n, S_j_ = n_j_*$\bar{y}$*/n*$\bar{y}$, (*j* = 1, 2, 3, 4……, *k*), and *D_jh_* is the interaction of the level of health resource allocation between the *j*, *h* regions.

### Kernel density estimation

Kernel density estimation is a nonparametric method for estimating the probability density function of a random variable, and is widely used in the study of spatially unbalanced distributions [24]. It represents the dynamic evolution process of the distribution pattern, location, degree of polarization and other characteristics of the data over time by means of a continuous density curve [25]. Kernel density estimation has some limitations. It is sensitive to the choice of bandwidth, which controls the width of the kernel density function, and if the bandwidth is not chosen correctly, it may result in an estimate that is too smooth or too volatile. The left and right shifts of the center point of the density curve indicate the decrease and increase of health resources in the measured region, the higher the peak of the curve means the trend of concentration of health resources is increasing, and the longer the trailing tail of the curve means the more significant the difference within the measured region. The formula is:

$$f(x)=\frac{1}{N_{h}}\sum_{i=1}^{N}K(\frac{X_{i}-x}{h})$$

where *N* is the number of observations, *h* is the bandwidth, *K*(·) is the kernel function, *X_i_* are the independent identically distributed sample values, and *x* is the sample mean.

There are more kinds of kernel density functions, among which the Gaussian kernel density function has the most applications, and this study uses the Gaussian kernel density function to analyze the dynamic evolution process of health resource allocation in sub-provincial cities in China. The calculation formula is:

$$f(x)=\frac{1}{\sqrt{2\pi }}\text{exp}\;(-\frac{x^{2}}{2})$$

### Statistical analysis

The data were recorded in Excel 2010, the health resource density index was calculated using Excel 2010, the Dagum Gini coefficient and the kernel density curve were calculated using SPSS 22.0, and the bandwidths of the kernel density curves were automatically calculated by the software in accordance with the ‘thumbs up rule’.

## Results

### Health resource density index of sub-provincial cities in China, 2022

In 2022, health resource density index of all six health resources in Shenzhen are the largest, and health resource density index of medical and health institutions, the number of beds, health technicians, licensed (assistant) physicians, registered nurses, and health expenditure reached 0.88, 11.06, 19.91, 7.95, 8.68, and 1,137.02, respectively, which indicates that the health resource allocation in Shenzhen was relatively well. Health resource density index of all six health resources in Harbin was the smallest, and health resource density index of medical and health institutions, the number of beds, health technicians, licensed (assistant) physicians, registered nurses, and health expenditure reached 0.21, 3.99, 3.61, 1.44, 1.61, and 50.33, respectively, indicating that the health resource allocation in Harbin was poor (Table 1).

**Table 1.** Health resource density index of sub-provincial cities in China, 2022.

Cities	Health expenditure	Medical and health institutions	Number of beds	Health technicians	Licensed (assistant) physicians	Registered nurses
Changchun	103.09	0.52	4.49	5.69	2.27	2.62
Harbin	50.33	0.21	3.99	3.61	1.44	1.61
Shenyang	97.97	0.47	7.25	8.59	3.33	4.06
Dalian	85.09	0.41	5.27	6.53	2.49	3.15
Shenzhen	1 137.02	0.88	11.06	19.91	7.95	8.68
Qingdao	131.67	0.81	6.29	9.17	3.82	4.11
Ningbo	198.15	0.51	4.82	9.00	3.70	3.88
Xiamen	383.56	0.80	7.57	14.25	5.85	6.25
Guangzhou	303.30	0.52	9.36	16.58	5.82	7.73
Nanjing	216.13	0.46	8.94	13.74	5.14	6.26
Hangzhou	182.33	0.40	6.43	10.28	3.98	4.56
Jinan	137.28	0.78	7.78	11.56	4.48	5.29
Wuhan	225.83	0.63	9.09	11.94	4.39	5.84
Chengdu	155.71	0.71	9.58	12.45	4.62	5.92
Xi’an	148.05	0.65	7.08	11.38	4.08	5.18

### Dagum Gini coefficient of health resource density index in sub-provincial cities in China, 2018–2022

The overall Gini coefficient of health expenditure increased from 0.373 in 2018 to 0.425 in 2022, the overall Gini coefficient of the number of beds increased from 0.155 in 2018 to 0.158 in 2022, and the overall Gini coefficient of licensed (assistant) physicians increased from 0.196 in 2018 to 0.201 in 2022, suggesting that the equity of health expenditure, number of beds and licensed (assistant) physicians will become progressively worse. The overall Gini coefficient of medical and health institutions decreased from 0.177 in 2018 to 0.174 in 2022, and the overall Gini coefficient of registered nurses decreased from 0.213 in 2018 to 0.204 in 2022, suggesting that equity of medical and health institutions and registered nurses is getting progressively better. The overall Gini coefficient of health expenditure was the largest and the overall Gini coefficient of number of beds was the smallest, indicating that the equity of health financial resources was the worst, the equity of health human resources was the second worst, and the equity of health physical resources was the best (Table 2).

**Table 2.** Dagum Gini coefficient of health resource density index in sub-provincial cities in China, 2018–2022.

Health resource	Years	Overall Gini coefficient	Intra-regional Gini coefficient			Inter-regional Gini coefficient			Contribution rate (%)		
			Northeastern region	Eastern region	Central and western region	Eastern & Northeastern region	Eastern & Central and western region	Northeastern & Central and western region	Intra-regional	Inter-regional	Hypervariable density
Health expenditure	2018	0.373	0.149	0.350	0.113	0.525	0.326	0.305	38.62	57.41	3.97
	2019	0.396	0.134	0.365	0.118	0.565	0.352	0.317	38.50	58.65	2.85
	2020	0.430	0.113	0.392	0.316	0.563	0.392	0.497	37.32	45.08	17.59
	2021	0.422	0.116	0.395	0.119	0.597	0.368	0.352	39.29	57.54	3.17
	2022	0.425	0.127	0.402	0.098	0.600	0.366	0.355	39.63	57.43	2.94
Medical and health institutions	2018	0.177	0.173	0.152	0.063	0.245	0.129	0.247	33.31	48.88	17.81
	2019	0.190	0.160	0.146	0.051	0.279	0.132	0.332	29.04	61.43	9.54
	2020	0.173	0.179	0.146	0.023	0.247	0.129	0.244	31.90	49.84	18.26
	2021	0.179	0.166	0.157	0.037	0.251	0.136	0.241	32.95	47.45	19.60
	2022	0.174	0.153	0.151	0.028	0.245	0.132	0.240	32.31	48.85	18.85
Number of beds	2018	0.155	0.122	0.122	0.095	0.168	0.154	0.254	30.01	58.84	11.16
	2019	0.155	0.115	0.112	0.086	0.182	0.150	0.274	27.62	63.24	9.14
	2020	0.155	0.117	0.130	0.069	0.202	0.122	0.243	31.49	55.50	13.01
	2021	0.155	0.127	0.132	0.060	0.206	0.118	0.240	31.86	54.79	13.36
	2022	0.158	0.126	0.134	0.065	0.217	0.115	0.243	31.89	54.26	13.86
Health technicians	2018	0.206	0.178	0.152	0.044	0.355	0.119	0.333	28.94	59.64	11.42
	2019	0.211	0.180	0.149	0.039	0.375	0.115	0.364	27.75	60.17	12.09
	2020	0.201	0.160	0.145	0.020	0.356	0.119	0.315	27.95	63.43	8.62
	2021	0.204	0.160	0.150	0.026	0.355	0.121	0.320	28.52	61.81	9.68
	2022	0.207	0.161	0.152	0.020	0.363	0.123	0.323	28.42	62.51	9.07
Licensed (assistant) physicians	2018	0.196	0.168	0.141	0.061	0.338	0.122	0.279	29.23	63.64	7.13
	2019	0.202	0.169	0.139	0.055	0.361	0.117	0.315	27.84	64.36	7.80
	2020	0.197	0.157	0.139	0.035	0.352	0.124	0.273	28.10	67.60	4.30
	2021	0.199	0.155	0.141	0.042	0.352	0.121	0.286	28.38	65.92	5.71
	2022	0.201	0.155	0.141	0.027	0.363	0.120	0.294	27.81	67.05	5.14
Registered nurses	2018	0.213	0.200	0.156	0.062	0.353	0.126	0.359	29.18	56.88	13.94
	2019	0.215	0.200	0.152	0.048	0.366	0.122	0.386	27.83	60.59	11.58
	2020	0.200	0.166	0.148	0.034	0.339	0.117	0.329	28.69	58.14	13.17
	2021	0.203	0.170	0.154	0.032	0.341	0.119	0.331	29.21	57.22	13.57
	2022	0.204	0.173	0.154	0.029	0.344	0.121	0.328	29.20	58.14	12.66

### Intra-regional differences, inter-regional differences, and contribution rate of regional differences of health resource density index in sub-provincial cities in China, 2018–2022

**Figure 2.**
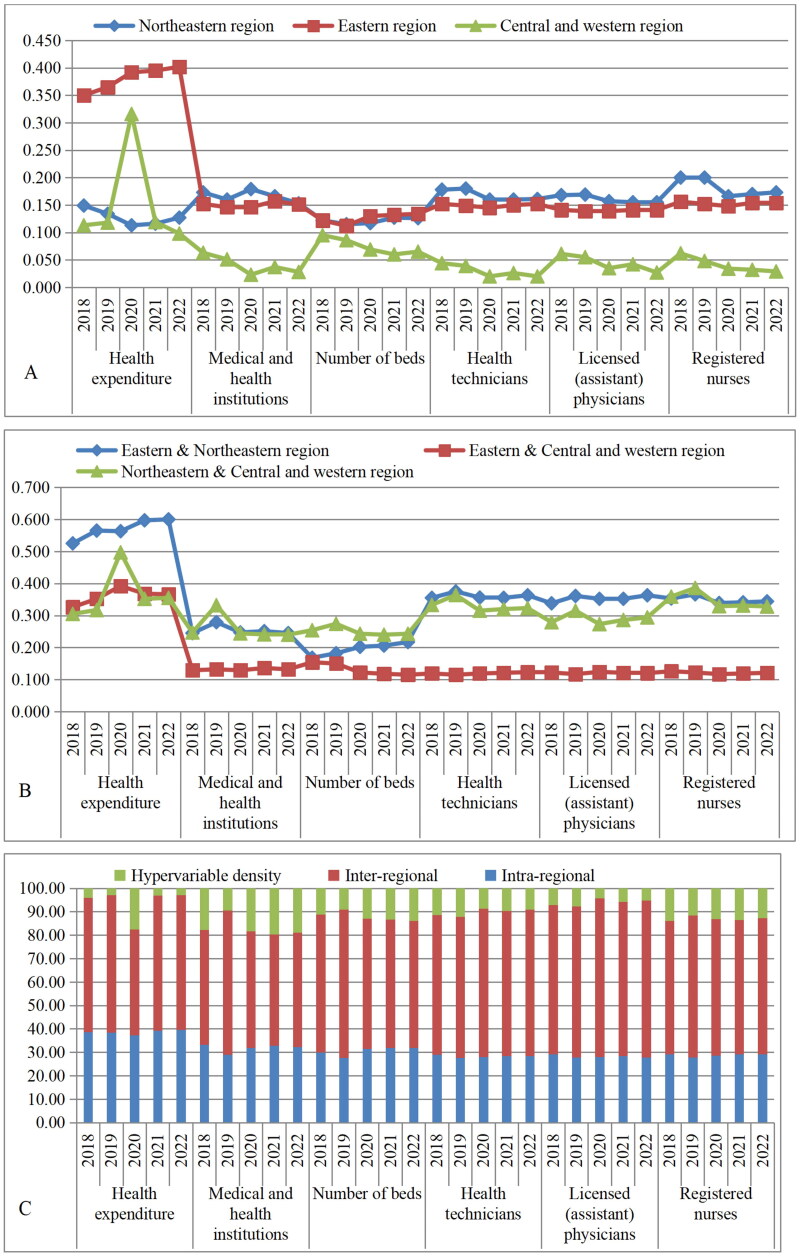
Intra-regional differences (a), inter-regional differences (B), and contribution rate of regional differences (C) of health resource density index in Sub-provincial cities in China, 2018–2022.

The differences in health expenditure are larger in the eastern region, and smaller in the central and western regions in terms of medical and health institutions, number of beds, health technicians, licensed (assistant) physicians and registered nurses. The differences in health expenditure and the number of beds in the eastern region are on an upward trend, and the differences in the number of beds in the northeastern region are on an upward trend, indicating that the intra-regional differences are getting progressively worse. The differences in the number of beds, health technicians, and registered nurses in the central and western regions show a decreasing trend, indicating that intra-regional differences are gradually improving (Figure 2A).

The difference in the health resource allocation in the eastern and central and western regions is relatively small, and the overall situation is relatively stable except for health expenditure, indicating that the health resources of the cities in the central and western regions have been developed faster with policy support, and the difference with the eastern region is relatively small. The difference in the allocation of health resources (except for the number of beds) was greater in the eastern and northeastern region, and the overall fluctuation was more obvious, indicating that there is a greater difference in the allocation of health resources between the northeastern region and the eastern region. The difference in the number of beds in the northeastern and central and western regions is greater, and the overall fluctuations are more obvious (Figure 2B).

The inter-regional contribution rate of the six health resources in sub-provincial cities in China was the main source of differences, followed by the intra-regional contribution rate and the lowest hypervariable density contribution rate, which was generally stable. The lowest value of the inter-regional contribution rate is 45.08% of health expenditure in 2020, and the highest value is 67.60% of the licensed (assistant) physicians in 2020. The intra-regional contribution rate fluctuates within the range of 27.62% to 39.63%. The hypervariable density contribution rate is relatively small and does not exceed 19.60% (Figure 2C).

### Kernel density distribution of health resource density index of health expenditure, medical and health institutions, the number of beds, health technicians, licensed (assistant) physicians, and registered nurses in sub-provincial cities in China, 2018–2022

**Figure 3.**
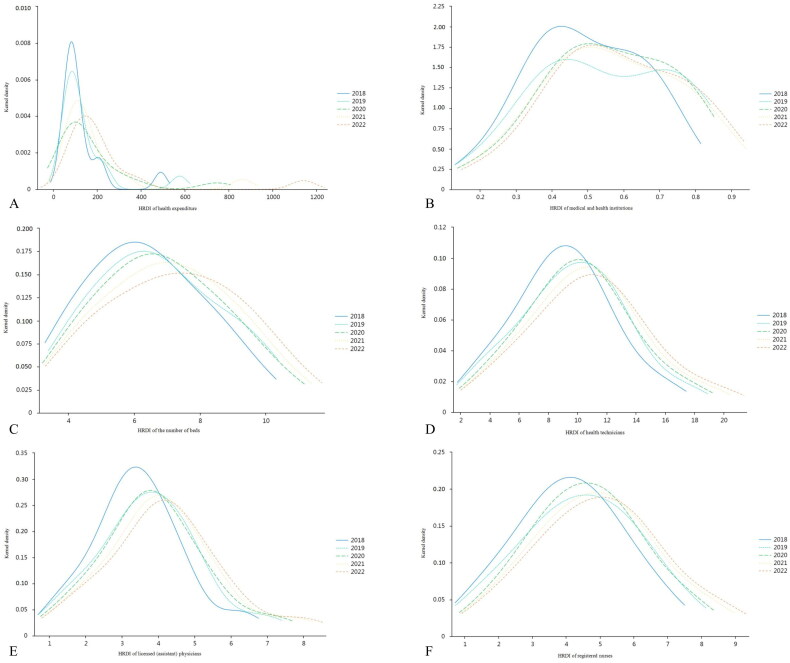
Kernel density distribution of health resource density index of health expenditure (a), medical and health institutions (B), the number of beds (C), health technicians (D), licensed (assistant) physicians (E), and registered nurses (F) in sub-provincial cities in China, 2018–2022.

The center point of the kernel density curve of health expenditure in sub-provincial cities in China is constantly shifting to the right, indicating that health expenditure in each city is increasing. The peak of the kernel density curve shows a downward trend, indicating that health expenditure is relatively decentralized. The right trailing of the kernel density curve is getting longer and longer, indicating that the differences in health expenditure between cities are becoming more and more significant. The kernel density curve of health expenditure has a multi-peaked distribution, indicating a low degree of equalization in the distribution of health expenditure (Figure 3A).

The center point of the kernel density curve of medical and health institutions in sub-provincial cities in China keeps moving to the right but slowly, indicating that medical and health institutions are growing slowly. The peak of the kernel density curve tends to decrease and then slowly increase, indicating that the differences between medical and health institutions are first becoming smaller and then gradually becoming larger. The kernel density curve has a smaller right trailing tail, indicating smaller differences in medical and health institutions among cities. The kernel density curves of medical and health institutions have a multi-peaked distribution, indicating that the equalization of medical and health institutions is low (Figure 3B).

The center point of the kernel density curve of the number of beds in sub-provincial cities in China keeps moving to the right, indicating that the number of beds continues to grow. The peak of the kernel density curve shows a downward trend, indicating that the number of beds is relatively decentralized. The right trailing of the kernel density curve is small, indicating that the differences in the number of beds between cities are small. The kernel density curve of the number of beds has a single-peaked distribution, indicating that the distribution of the number of beds is relatively balanced (Figure 3C).

The center point of the kernel density curve of health technicians in sub-provincial cities in China is constantly shifting to the right, indicating that health technicians in each city continue to grow. The peaks of the kernel density curves show a downward trend, indicating that the health technicians are relatively decentralized. The right trailing of the kernel density curve is small, indicating that the differences in health technicians between cities are small. The kernel density curve of health technicians has a single-peak distribution, indicating that the distribution of health technicians is relatively balanced (Figure 3D).

The center point of the kernel density curve of licensed (assistant) physicians in sub-provincial cities in China is constantly shifting to the right, indicating that licensed (assistant) physicians in each city continue to grow. The peaks of the kernel density curves show a decreasing trend, indicating that licensed (assistant) physicians are relatively dispersed. The right trailing of the kernel density curve is small, indicating that there is less difference in licensed (assistant) physicians between cities. The kernel density curve of licensed (assistant) physicians has a single-peaked distribution, indicating that the distribution of licensed (assistant) physicians is relatively balanced (Figure 3E).

The center point of the kernel density curve of registered nurses in sub-provincial cities in China is constantly shifting to the right, indicating that registered nurses continue to grow in each city. The peaks of the kernel density curves show a decreasing trend, indicating that registered nurses are relatively decentralized. The right trailing of the kernel density curve is small, indicating that there is less difference in registered nurses between cities. The kernel density curve of registered nurses has a single-peaked distribution, indicating a relatively equal distribution of registered nurses (Figure 3F).

## Discussion

The center point of the kernel density curve of health resources in sub-provincial cities in China is constantly shifting to the right, indicating that health resources in sub-provincial cities in China continue to grow. The level of health resource allocation, which combines population and geographical factors, has been gradually increasing, and can better meet the multi-level and diversified health care needs of the people [26]. We believe there are three reasons for this. First, sub-provincial cities have attached importance to health resource development and provided policy support. Sub-provincial cities have formulated health policies to promote the development of health resources, attracted more health talents through the construction of national medical centers and national regional medical centers, and improved the hardware construction of medical institutions through medical service capacity enhancement projects, thus promoting the rapid growth of health resources as a whole [27]. Second, as a sub-provincial city, it gathers more population and economy, which is conducive to building it into a highland for health. Sub-provincial cities have larger populations and higher levels of economic development, which leads to better room for growth in both population factors and health funding inputs, thus promoting the growth of health resources [28]. Third, the medical market in sub-provincial cities is active, and social medical services are better developed. The medical market in sub-provincial cities is active, with many medical needs and strong people’s willingness and ability to pay, thus promoting the development of medical resources [29]. The social medical services have developed vigorously, playing a very important complementary role in the medical market, thus promoting the gradual improvement of the allocation of medical resources in sub-provincial cities. Therefore, sub-provincial cities should attach great importance to the growth of health resources, strengthen the analysis of key data used for health resource mobilization and coordinated decision-making [30], continuously increase investment in health resources [31], and improve the quality and efficiency of health resources [32]. It is also necessary to implement the functional positioning of public and private hospitals and to promote a gradual increase in the level of health resource allocation, so as to make better use of the driving role of high-quality medical resources [33].

The Dagum Gini coefficient of health resource allocation in sub-provincial cities in China is within 0.2, and the right trailing of the kernel density curve is small (except for health expenditure), which indicates that the overall balance of health resource allocation in sub-provincial cities in China is better, but the differences in health expenditure are significant. We believe there are two reasons for this. On the one hand, health expenditure is strongly influenced by the level of economic development, which leads to significant differences among sub-provincial cities. Theoretically, the higher the level of economic development, the more the government can invest in health [34]. Although the sub-provincial cities are at one administrative level, they are located in different population and geographical locations, which leads to different levels of economic development, which affects the level of health expenditure [35].The Dagum Gini coefficient of health expenditure in 2020 reached a maximum value of 0.430, which may have been affected by the impact of the COVID-19 pandemic 2019 on the economy since December. On the other hand, health physical resources and health human resources are relatively more stable and better balanced overall. Health physical resources are relatively more administratively constrained and can be built up quickly with administrative intervention, resulting in relatively better stability. The Dagum Gini coefficient of medical and health institutions is within 0.2, but the kernel density curve has a multi-peak distribution, which may be caused by the adjustment of administrative divisions leading to the adjustment of medical and health institutions. Although human resources for health are characterized by high mobility, the "siphoning effect" of sub-provincial cities on health resources ensures that their own human resources for health are replenished, and the overall balance is good. Therefore, sub-provincial cities must continue to promote economic development, thereby stabilizing the level of health expenditure and promoting the development of health resources to better meet the people’s needs [36]. It is also necessary to emphasize the balanced development of health resources, rationally regulate the size of medical institutions, and improve the quality of medical services [37], so as to improve the quality of the health resource allocation.

The lowest value of the inter-regional contribution rate is 45.08%, which is still 7 percentage points higher than the intra-regional contribution rate in the same period, indicating that inter-regional differences are the main cause of differences in health resource allocation in sub-provincial cities in China. We believe there are three reasons for this. First, the uneven level of regional economic development has led to greater differences in health resource allocation [38]. In 2022, the highest per capita GDP in the eastern region, Shenzhen, reaches RMB 18,390.37, while the highest per capita GDP in the northeastern and central and western regions are Dalian (RMB 11,194.93) and Wuhan (RMB 13,732.03), respectively, with a clear gap in the level of economic development. The eastern region has a higher level of economic development, more health expenditure, and is overall richer in health resource allocation [39]. The central and western regions are constrained by factors such as a weaker historical foundation and a more distant geographic location, and are overall in a poorer position in health resource allocation. The northeastern region is an old industrial base, and its economic level has stagnated or even regressed, affecting the further development of health resources. Although the central and western regions and the northeastern region are actively developing health resources, there is still a large gap with the eastern region [40]. Second, differences in medical needs have led to greater disparities in the allocation of medical resources among regions. In the eastern region, the population is more concentrated, the ability to pay for medical insurance is stronger, and the people’s awareness of medical care is higher, which leads to a stronger demand for medical care and promotes the development of medical resources. The northeastern, central and western regions are relatively weak in terms of their ability and willingness to pay for medical care, and are influenced by the traditional concept of medical care, so that the demand for medical care plays a relatively weaker role in promoting the development of health resources [41]. Third, medical facilities and equipment, medical technology and service quality all work together to increase health resources. In the eastern region, medical facilities and equipment are more advanced, medical technology and service quality are generally higher, and the overall quality of health resources is better, which promotes the development of health resources. Therefore, the northeastern, central and western regions should continue to develop their economies, popularize health education and knowledge, and continue to learn from the experience of the eastern region in developing high-quality medical resources, so as to gradually narrow the gap with the eastern region and improve the balance in the allocation of health resources among regions [42]. The eastern region can strengthen its health counterpart support to the central and western regions and improve the quality and level of health services, thereby promoting the better utilization of high-quality health resources for the benefit of the people [43].

The difference in the allocation of health resources between the eastern & central and western regions is smaller, indicating that the health resources of the sub-provincial cities in the central and western regions have been developed faster with policy support, and the difference with the eastern region is smaller. There are fewer sub-provincial cities in the central and western regions, but they are all more developed cities in terms of social, economic and population factors, and they have gathered more medical and health institutions, large medical institutions and many medical personnel in the province, with a higher level of development and better allocation of health resources overall [44]. Although the eastern region as a whole has a higher level of economic development, some of the sub-provincial cities have relatively small populations (In 2022, the resident population of Xiamen in the eastern region is the lowest among sub-provincial cities at 5,308,000, and that of Chengdu in the central and western regions is the highest at 21,268,000.), resulting in insufficient concentration of health resources, and they are unable to catch up with the sub-provincial cities in the central and western regions in terms of health resource allocation [45]. The relatively large differences in the allocation of health resources between the northeastern region and the eastern, central and western regions indicate that the northeastern region has been slow to develop its health resources and that the differences with other regions are gradually widening. The northeastern region is an old industrial base, and with the development of economic transformation, the economy has fallen into a state of stagnation or even regression, making it difficult to provide adequate health expenditure for the development of health resources. The population of sub-provincial cities in the northeastern region is not superior to that of other regions, making it difficult for population concentration to have a greater impact on the allocation of health resources, thus resulting in the slow development of health resources. Therefore, the northeastern region should vigorously develop its economy and promote economic recovery and development through the revitalization of old industrial bases and other policy support, while at the same time increasing investment in the development of health resources, so as to promote the growth of health resources [46]. The eastern and central and western regions should promote the expansion and sinking of high-quality medical resources, improve the service system of high-quality health resources by cities with abundant health resources helping grass-roots areas with weak health resources, and promote the high-quality development of health resources [47].

### Limitations

Although we used Dagum Gini coefficient and kernel density estimation based on health resource density index to analyze the regional disparities and trends in health resource allocation in sub-provincial cities in China, this study still has some limitations. First, we analyzed the regional disparities and trends in health resource allocation in sub-provincial cities in China, but did not cover the output of health resources and the relationship between the input and output of health resources. In future research, we can comprehensively analyze the equity and efficiency of the allocation of health resources in sub-provincial cities, the geospatial disparities in health resources, and so on. Second, although we analyzed the contribution rate of intra- and inter-regional differences, we did not further analyze the influencing factors of health resource allocation. In future research, we can comprehensively consider the impact of factors such as population aging, residents’ actual demand for health care, and policy implementation on health resource allocation. Third, although we have comprehensively considered the impact of population and geographic factors on health resources, we did not address regional economic factors. In future research, we can further analyze the urban-rural differences in health resource allocation in sub-provincial cities, the coupling and coordination of health resources and economic development, and so on. In terms of data selection, research can be explored in conjunction with important years of healthcare reform, and in terms of research methodology, geospatial analysis under the condition of multiple influencing factors can be explored, all of which can help to express the allocation of health resources in sub-provincial cities in a clearer and more accurate way.

## Conclusions

In order to promote the coordinated regional development of health resources in sub-provincial cities in China, we used the Dagum Gini coefficient and kernel density estimation based on health resource density index to analyze the regional disparities and trends in health resource allocation in sub-provincial cities in China. It was found that the overall Gini coefficient of health expenditure is the largest, above 0.373, and the overall Gini coefficient of the number of beds is the smallest, below 0.158. The differences in health expenditure are larger in the eastern region, and smaller in the central and western regions in terms of medical and health institutions, number of beds, health technicians, licensed (assistant) physicians and registered nurses. The inter-regional contribution rate is the main source of differences, with the lowest value of the inter-regional contribution rate being 45.08% of health expenditure in 2020 and the highest value being 67.60% of licensed (assistant) physicians in 2020. The center point of the kernel density curve is constantly shifting to the right, indicating that health resources are growing in each city. The longer and longer right trailing tail of the health expenditure kernel density curve indicates a significant difference in health expenditure. The kernel density curves of health expenditure and medical and health institutions have a multi-peaked distribution, indicating a low degree of equalization, while the rest of the distribution of health resources is relatively more balanced. We suggest that sub-provincial cities should continue to promote economic development, continue to strengthen their investment in health resources, improve the quality and efficiency of health resources, and promote the development of health resources to better meet the people’s needs for medical care. Health expenditures should set planning targets with reference to better-balanced resources, such as health technicians, and increase support for the northeastern and central and western regions through central financial transfers to improve the balance of health investments. The eastern region should strengthen its health counterpart support to the central and western regions, so as to enhance the quality and level of health services. The northeastern region should increase targeted investment in the development of health resources and promote the growth of health resources. The northeastern and central and western regions should continue to learn from the experience of the eastern region in the development of high-quality medical resources, and gradually narrow the gap with the eastern region, so as to improve the balance in the allocation of health resources between regions.

## Data Availability

The data that support the findings of this study are available in China Health Statistics Yearbook at http://www.nhc.gov.cn/mohwsbwstjxxzx/tjtjnj/tjsj_list.shtml, reference number 2018–2022. The data that support the findings of this study are available on request from the corresponding author, MHZ.
